# Prevalence and factors associated with knee osteoarthritis among middle-aged and elderly individuals in rural Tianjin: a population-based cross-sectional study

**DOI:** 10.1186/s13018-023-03742-4

**Published:** 2023-04-01

**Authors:** Shuqing Ji, Li Liu, Jiwei Li, Guohua Zhao, Yana Cai, Yanan Dong, Jinghua Wang, Shengguang Wu

**Affiliations:** 1 Department of Orthopedics, Tianjin Jizhou Hospital of Traditional Chinese Medicine, Tianjin, 301900 China; 2 Department of Pharmacy, Tianjin Jizhou People’s Hospital, Tianjin, 301900 China; 3 Department of Emergency, Tianjin Jizhou People’s Hospital, Tianjin, 301900 China; 4 Department of Nursing, Tianjin Jizhou People’s Hospital, Tianjin, 301900 China; 5 Laboratory of Epidemiology, Tianjin Neurological Institute, Tianjin, 300052 China; 6 Department of Surgery, Tianjin Jizhou Hospital of Traditional Chinese Medicine, 19 Yuyangnan Road, Tianjin, 301900 China

**Keywords:** Knee osteoarthritis, Epidemiology, Prevalence, Risk factors, Population-based cross-sectional study

## Abstract

**Background:**

The prevalence of osteoarthritis has been investigated in many countries and regions. Considering the wide differences in ethnicity, socioeconomic status, environmental factors, and lifestyle patterns, our study aimed to report the prevalence of knee osteoarthritis (KOA) and its associated factors in rural areas of Tianjin.

**Methods:**

This population-based cross-sectional study was conducted between June and August 2020. KOA was diagnosed according to the 1995 American College of Rheumatology criteria. Information on participants’ age, years of education, BMI, smoking and drinking status, sleep quality, and frequency of walking were collected. Multivariate logistic regression analysis was used to analyze factors influencing KOA.

**Results:**

This study included 3924 participants (1950 male and 1974 female); the mean age of all participants was 58.53 years. In total, 404 patients were diagnosed with KOA, and the overall prevalence of KOA was 10.3%. The prevalence of KOA was higher in women than in men (14.1% vs. 6.5%). The risk of KOA in women was 1.764 times higher than that in men. The risk of KOA increased following the increasement of age. There was higher risk of KOA in participants who walked frequently than in participants who walked infrequently (OR = 1.572); in participants with overweight than in participants with normal weight (OR = 1.509), in participants with average sleep quality (OR = 1.677) and those with perceived poor sleep quality (OR = 1.978), respectively, than participants with satisfactory sleep quality, and in postmenopausal women than in non-menopausal women (OR = 4.12). The risk of KOA in participants with an elementary level was lower (0.619 times) than participants with illiteracy. In addition, the results of gender subgroup analysis showed that in male, age, obesity, frequent walking and sleep quality were independent factors associated with KOA; while in female, age, BMI, education level, sleep quality, frequent walking and whether menopausal were independent factors associated with KOA (P < 0.05).

**Conclusion:**

The results of our population-based cross-sectional study showed that sex, age, educational level, BMI, sleep quality, and frequent walking were independent influencing factors for KOA, and the influencing factors for KOA differed between the sexes. In order to reduce the disease burden of KOA and the harm to the health of middle-aged and elderly people, the risk factors related to the control of KOA should be identified as much as possible.

*Trial registration*: ChiCTR2100050140.

## Introduction

Osteoarthritis (OA) is a chronic degenerative joint disease that comprises part of the aging process. It is characterized by the loss and degradation of articular cartilage in addition to synovial inflammation, leading to joint stiffness, swelling, and loss of mobility [1]. It can also cause serious localized pain, deprive joint function, and eventually lead to disabilities. This leads to a high medical and economic burden on society owing to the increased proportion of the elderly population. The prevalence of OA is expected to increase by about 40%, making OA the fourth most common cause of disability worldwide in 2020 [2, 3]. In 2010, the global age-standardized prevalence of knee OA was 3.8% [4]. OA mainly affects the joints of the knees, hips, hands, facets, and feet, and because of the higher vulnerability of the knee to direct and indirect trauma, along with the high load supported by this joint, the knee is the joint most frequently affected by OA: previous data showed that knee OA (KOA) accounts for 83% of the total OA burden [5]. In China, the prevalence of KOA in China was 21.51% [6].

Understanding the risk factors for KOA is important for managing health among adults, particularly in the elderly population. Several studies investigating the risk factors for KOA have been conducted in the US, UK, and other developed countries [7–9]. Moreover, some studies on KOA have been conducted in cities in China [10, 11]. However, considering substantial differences in race, socioeconomic status, environmental factors, and lifestyle patterns, these findings have limited implications for rural populations.

The aim of this study was to investigate the prevalence and risk factors for KOA in a rural area of Tianjin China.

## Patients and methods

This population-based cross-sectional study was conducted between June and August 2020. The study participants were drawn using a random cluster stratified sampling method from the resident population in the rural areas of Tianjin.

Four towns in the Jizhou District of Tianjin were randomly selected, with one village selected from each town. All participants aged at least 40 years were included in the study. Inclusion criteria were (1) age ≥ 40 years and (2) permanent resident with registered household registrations in the Jizhou District, Tianjin. Exclusion criteria were (1) age less than 40 years; (2) no local household registration; (3) unwilling to cooperate with the study or unwilling to sign an informed consent form; (4) secondary arthritis (including congenital or developmental diseases); (5) inflammatory arthritis (systemic lupus erythematosus, rheumatoid arthritis, rheumatoid arthritis, gouty arthritis, hemophilic arthritis, etc.); and (6) major mental illness.

All study protocols were approved by Chinese Ethics Committee of Registering Clinical Trials (ChiECRCT20210021), and all participants provided informed consent.

### Collection of patient data

Participant information, including sex, date of birth, education level, sleep quality, and walking, was obtained from existing records. Participants were divided into four age groups: 40–55 years, 55–64 years, 65–74 years, ≥ 75 years. Educational level was divided into the following categories: illiterate, primary, elementary, high school, and college.

### Definition of risk factors

Personal lifestyle characteristics, including smoking and drinking. Participants were divided into smoking, quitting, and never smoking groups by smoking status; smoking > 1 cigarette per day for at least 1 year, quitting smoking was defined as quitting smoking for at least half a year. Participants were divided into drinking, abstaining, and never drinking groups by drinking status. Drinking was defined as drinking more than 500 g per week for at least one year, abstaining was defined as quitting drinking for at least half year. Body mass index ([BMI] = weight (kg)/height [m]^2^) was used as a measure of relative body weight, with BMI < 24.0 kg/m^2^ defined as normal weight, 24.0 kg/m ≤ BMI < 28.0 kg/m^2^ as overweight, and BMI ≥ 28.0 kg/m^2^ as obese. Walking was defined as walking two miles or more at least once a week [12].

### Diagnosis of KOA

The 1995 American College of Rheumatology clinical diagnostic criteria for KOA are as follows [13]: (1) repeated knee pain in the past month; (2) age ≥ 50 years; (3) morning stiffness ≥ 30 min; (4) crepitus on motion; and (5) radiographs (standing or weight-bearing) show joint space narrowing, subchondral sclerosis, and/or cystic degeneration, along with articular edge osteophyte formation. KOA can be diagnosed if the above criteria 1, 2, 3, and 4 are met at the same time, or if the criteria 1, 5 are met.

### Statistical analysis

Continuous variables are expressed as the mean ± standard deviation. Age, years of education, BMI, and sleep duration between the two groups of men and women were tested using the independent samples T test. Categorical variables such as groups for age, BMI, education level, smoking, drinking, sleep quality, and frequent walking were tested using the chi-squared test; multivariate logistic regression analysis was used to analyze the risk factors affecting KOA. The selection criteria for the inclusion of variables in the multivariate analysis was P < 0.25 for independent variables in the univariate analysis [14]. Adjusted variables, including sex, age, BMI, education level, smoking status, alcohol consumption, sleep status, and frequent walking, were included in the multivariate analysis of the entire population. Statistical significance was set at P < 0.05. Statistical analysis was performed using SPSS 25.0 statistical software.

## Results

### Demographical characteristics of participants

There were 3924 study participants, including 1950 male and 1974 female participants. The average age of all participants was 58.53 years, of which 1393 (35.7%) were aged less than 55 years and 1344 (34.4%) were aged 55–64 years. Of all participants, 12.1% had a high school and over education, 41.3% had a normal BMI, 16.2% had obesity, 79.7% reported satisfactory sleep quality, 23.2% were current drinkers, and 24.4% were current smokers, 28.7% often walk (Table 1).

**Table 1:** Baseline characteristics of male and female

Characteristics	Male	Female	Total
Cases, n (%)	1950 (49.7)	1974 (50.3)	3924 (100)
Age, year, mean (SD)	58.44 (9.25)	58.62 (9.16)	58.53 (9.20)
Age group, n (%)			
< 55 years	697 (35.9)	696 (35.5)	1393 (35.7)
55–64 years	663 (34.1)	681 (34.7)	1344 (34.4)
65–74 years	525 (27.0)	504 (25.7)	1029 (26.4)
≥ 75 years	58 (3.0)	81 (4.1)	139 (3.6)
Education, means (SD), years	7.59 (2.82)	6.60 (3.49)	7.10 (3.21)
Education, n (%)			
Illiterate	68 (3.5)	281 (14.2)	349 (8.9)
Primary level	542 (27.8)	560 (28.4)	1102 (28.1)
Elementary level	1075 (55.2)	914 (46.3)	1989 (50.7)
High school	240 (12.3)	200 (10.1)	440 (11.2)
≥ College	23 (1.2)	17 (0.9)	40 (1.0)
Smoking, n (%)			
Never smoking	885 (45.5)	1919 (97.4)	2804 (71.6)
Ex-smoker	147 (7.6)	10 (0.5)	157 (4.0)
Current smoker	914 (47.0)	41 (2.1)	955 (24.4)
Drinking, n (%)			
Never drinking	982 (50.5)	1945 (98.8)	2927 (74.8)
Ex-drinker	78 (4.0)	0 (0.0)	78 (2.0)
Current drinker	886 (45.5)	24 (1.2)	910 (23.2)
BMI, means (SD), kg/m^2^	24.49 (3.53)	25.14 (6.50)	24.82 (5.25)
BMI group, n (%)			
Normal	889 (46.1)	713 (36.6)	1602 (41.3)
Over weight	786 (40.8)	863 (44.3)	1649 (42.5)
Obesity	253 (13.1)	374 (19.2)	627 (16.2)
Sleeping time, means (SD), hours	7.64 (1.03)	7.44 (0.94)	7.54 (0.99)
Sleep situation, n (%)			
Satisfy	1672 (86.4)	1435 (73.0)	3107 (79.7)
Not bad	154 (8.0)	340 (17.3)	494 (12.7)
Insufficient	109 (5.6)	190 (9.7)	299 (7.7)
Often walk			
Yes	439 (23.0)	668 (34.3)	1107 (28.7)
No	1468 (77.0)	1280 (65.7)	2748 (71.3)

A total of 404 participants developed KOA, including 126 male (accounted for 31.2%). Of these patients, the mean age was 62.71 years, and 85.1% of the patients were older than 55 years, the average years of education was 6.24 years; 83.4% of patients were never smokers, 82.9% were never drinkers, and 70% were overweight or obese. The proportion of patients with satisfactory sleep quality was 65.1% (Table 2).

**Table 2:** Baseline characteristics of KOA

Characteristics	Male	Female	Total
Cases, n (%)	126 (31.2)	278 (68.8)	404 (100)
Age, year, mean (SD)	62.04 (7.46)	63.01 (6.97)	62.71 (7.13)
Age group, n (%)			
< 55 years	24 (19.0)	36 (12.9)	60 (14.9)
55–64 years	54 (42.9)	122 (43.9)	176 (43.6)
65–74 years	40 (31.7)	102 (36.7)	142 (35.1)
≥ 75 years	8 (6.3)	18 (6.5)	26 (6.4)
Education, means (SD), years	7.58 (2.66)	5.61 (3.78)	6.24 (3.57)
Education, n (%)			
Illiterate	3 (2.4)	63 (22.7)	66 (16.3)
Primary level	38 (30.2)	96 (34.5)	134 (33.2)
Elementary level	63 (5.0)	86 (30.9)	149 (36.9)
High school	22 (17.5)	33 (11.9)	55 (13.6)
≥ College	0 (0)	0 (0)	0 (0)
Smoking, n (%)			
Never smoking	65 (51.6)	272 (97.8)	337 (83.4)
Ex-smoker	9 (7.1)	2 (0.7)	11 (2.7)
Current smoker	52 (41.3)	4 (1.4)	56 (13.9)
Drinking, n (%)			
Never drinking	62 (49.2)	272 (98.2)	334 (82.9)
Ex-drinker	7 (5.6)	0 (0)	7 (1.7)
Current drinker	57 (45.2)	5 (1.8)	62 (15.4)
BMI, means (SD), kg/m^2^	25.25 (4.49)	26.13 (3.98)	25.86 (4.16)
BMI group, n (%)			
Normal	46 (37.1)	74 (26.9)	120 (30.1)
Over weight	53 (42.7)	128 (46.5)	181 (45.4)
Obesity	25 (20.2)	73 (26.5)	98 (24.6)
Sleeping time, means (SD), hours	7.58 (0.81)	7.14 (1.27)	7.28 (1.16)
Sleep situation, n (%)			
Satisfy	96 (76.8)	165 (59.9)	261 (65.1)
Not bad	17 (13.6)	68 (24.6)	85 (21.2)
Insufficient	12 (9.6)	43 (15.6)	55 (13.7)
Often walk			
Yes	53 (42.1)	112 (40.3)	165 (40.8)
No	166 (59.7)	73 (57.9)	239 (59.2)

### Prevalence of KOA and associated factors in the univariate analysis

In all participants, 404 (10.3%) were diagnosed with KOA. The prevalence of KOA was higher in women than in men (14.1% vs. 6.5%; P < 0.001), in frequent walking than in those with infrequent walking (14.9% vs. 8.7%; P < 0.001). The prevalence of KOA associated with age groups, BMI groups, and sleep quality, and smoking and driking status (all P < 0.001; Table 3).

**Table 3:** Association of various factors with the risk of knee osteoarthritis in this study

Variables	Non-OA(n)	OA(n)	Prevalence rate (%)	P value
Sex				< 0.001
Male	1824	126	6.5	
Female	1696	278	14.1	
Age group				< 0.001
< 55 years	1333	60	4.3	
55–64 years	1168	176	13.1	
65–74 years	887	142	13.8	
≥ 75 years	113	26	19.1	
Education				< 0.001
Illiterate	283	66	18.9	
Primary level	968	134	12.2	
Elementary level	1840	149	7.0	
High school	385	55	12.5	
≥ College	40	0	0.0	
Smoking				< 0.001
Never smoking	2467	337	12.0	
Ex-smoker	146	11	7.0	
Current smoker	899	56	5.9	
Drinking				< 0.001
Never drinking	2593	334	11.4	
Ex-drinker	71	7	9.0	
Current drinker	848	62	6.8	
BMI group				< 0.001
Normal	1482	120	7.5	
Over weight	1468	181	11.0	
Obesity	529	98	15.6	
Sleep situation				< 0.001
Satisfy	2846	261	8.4	
Not bad	409	85	17.2	
Insufficient	244	55	18.4	
Often walk				< 0.001
Yes	942	165	14.9	
No	2509	239	8.7	

### Associated factors for KOA in the multivariate analysis

Age, BMI, sleep quality, educational background, and frequent walking were significantly associated with KOA (P < 0.05). The risk of KOA was 1.764 times higher in women than in men (95%CI 1.291–2.409), and the risk of KOA in participants aged 55–64, 65–74, and over 75 years was 2.913 times (95%CI 2.123–3.997), 2.784 times (95%CI 1.959–3.956), and 3.439 times higher than in participants aged under 55 years (95%CI 1.999–5.917), respectively. The risk of KOA was 1.572 times higher (95% CI 1.255–1.968) in participants who walked frequently than in participants who walked infrequently; 1.509 times (95%CI 1.172–1.943) and 2.235 times higher (95%CI 1.653–3.021) in participants with overweight and obesity, respectively, than in participants with normal weight; and 1.677 times (95%CI 1.265–2.224) and 1.978 times higher (95%CI 1.410–2.776) in participants with average sleep quality and perceived poor sleep quality, respectively, than in participants with satisfactory sleep quality. In contrast, the risk of KOA was lower (0.619 times; 95%CI 0.430–0.891) in participants with an elementary level than in participants who were illiterate. Results of the multivariate analysis are reported in Table 4.

**Table 4:** Multivariable logistic regression of the risk factors for knee osteoarthritis in the entire study population (both sexes combined)

Variables	Reference	OR	95% CI	P value
Sex	Male			
Female		1.764	1.291–2.409	< 0.001
Age	< 55 years			
55–64 years		2.913	2.123–3.997	< 0.001
65–74 years		2.784	1.959–3.956	< 0.001
≥ 75 years		3.439	1.999–5.917	< 0.001
BMI	Normal			
Over weight		1.509	1.172–1.943	0.001
Obesity		2.235	1.653–3.021	< 0.001
Sleep situation	Satisfy			
Not bad		1.677	1.265–2.224	< 0.001
Insufficient		1.978	1.410–2.776	< 0.001
Often walk	No			
Yes		1.572	1.255–1.968	< 0.001
Education	Illiterate			
Primary level		0.805	0.570–1.137	0.219
Elementary level		0.619	0.430–0.891	0.010
High school		0.846	0.549–1.303	0.448
≥ College		–	–	–
Smoking	Never smoking			
Ex-smoker		0.660	0.319–1.368	0.264
Current smoker		0.717	0.485–1.059	0.094
Drinking	Never drinking			
Ex-drinker		1.568	0.653–3.768	0.314
Current drinker		1.280	0.867–1.890	0.215

### Associated factors in the subgroup analysis by sex

In the subgroup analysis, for male participants, age, frequent walking, BMI, education level and sleep quality; age, frequent walking, BMI and sleep quality were independent influencing factors for KOA (P < 0.05). The risk of KOA was 2.460 times (95%CI 1.471– 4.115), 2.102 times (95%CI 1.177–3.755), and 4.258 times higher (95%CI 1.691–10.725) in participants aged 55–64, 65–74, and over 75 years than in participants aged under 55 years; 2.191 times higher (95%CI 1.477–3.250) in participants with frequent walking than in participants with infrequent walking; and 2.351 times greater (95%CI 1.218–4,539) in participants with perceived poor sleep quality, respectively, than in participants with satisfactory sleep quality; 2.049 times higher (95%CI 1.195–3.513) in participants with obesity than in participants with normal weight (all P < 0.05; Table 5).

**Table 5:** Multivariate logistic regression of risk factors for knee osteoarthritis in men

Variables	Reference	OR	95% CI	P value
BMI	Normal			
Over weight		1.347	0.885–2.050	0.165
Obesity		2.049	1.195–3.513	0.009
Sleep situation	Satisfy			
Not bad		1.620	0.908–2.892	0.103
Insufficient		2.351	1.218–4.539	0.011
Education	Illiterate			
Primary level		2.636	0.611–11.377	0.194
Elementary level		2.514	0.580–10.891	0.218
High school		3.473	0.767–15.721	0.106
≥ College		–	–	–
Age	< 55 years			
55–64 years		2.460	1.471–4.115	0.001
65–74 years		2.102	1.177–3.755	0.012
≥ 75 years		4.258	1.691–10.725	0.002
Often walk	No			
Yes		2.191	1.477–3.250	< 0.001

After adjusting for age, education level, BMI, frequent walking, menopause, and sleep quality among all female participants, age, BMI, education level, sleep quality, and menopausal status age were independent influencing factors for KOA (P < 0.05). The risk of KOA was 1.677 times higher (95%CI 1.058–2.657) in participants aged 55–64 years than in participants aged under 55 years; 1.530 times (95%CI 1.115–2.099) and 2.201 times higher (95%CI 1.527–3.172) in participants with overweight and obesity, respectively, than in participants with normal weight; 1.664 times (95%CI 1.118–2.275) and 1.811 times higher (95%CI 1.221–2.687) in participants with average sleep quality and those with perceived poor sleep quality, respectively, than in participants with satisfactory sleep quality; 1.333 times higher (95%CI 1.014–1.753) in participants with frequent walking than in participants with infrequent walking; and 4.120 times higher (95%CI 2.116–8.021) in postmenopausal women that in non-menopausal women. The risk of KOA among women in the Elementary level was 0.557 times lower than that of the illiterate group(95%CI 0.371–0.838). Results of the sex-specific analysis are reported in Table 6.

**Table 6:** Multivariate logistic regression of risk factors for knee osteoarthritis in women

Variables	Reference	OR	95% CI	P value
BMI	Normal			
Over weight		1.530	1.115–2.099	0.008
Obesity		2.201	1.527–3.172	< 0.001
Sleep situation	Satisfy			
Not bad		1.644	1.118–2.275	0.003
Insufficient		1.811	1.221–2.687	0.003
Menopause	No menopause			
Yes		4.120	2.116–8.021	< 0.001
Education	Illiterate			
Primary level		0.768	0.528–1.118	0.168
Elementary level		0.557	0.371–0.838	0.005
High school		0.724	0.433–1.212	0.219
≥ College		–	–	–
Age	< 55 years			
55–64 years		1.677	1.058–2.657	0.028
65–74 years		1.614	0.982–2.653	0.059
≥ 75 years		1.699	0.840–3.439	0.140
Often walk	No			
Yes		1.333	1.014–1.753	0.040

## Discussion

Many countries and regions have investigated the prevalence of osteoarthritis. Considering the large differences between different ethnic groups, socioeconomic status, environmental factors, and lifestyle patterns. We report the prevalence of KOA and its associated influencing factors in rural areas of Tianjin. The overall prevalence of KOA in the study population was 10.3%, and the risk of KOA in women was 1.764 times higher than in men. Moreover, the risk of KOA increased with increasing age, increasing BMI, a decline of sleep quality, and frequent walking, while the risk of KOA was lower with increased education level. In conclusion, sex, age, education level, BMI, sleep quality, and walking frequency were independent factors influencing KOA. In addition, factors influencing knee osteoarthritis exhibit sex-specific differences. For example, the risk of KOA in postmenopausal women is 4.12 times higher than that in premenopausal women.

In a Nigerian study, the prevalence of KOA was approximately 15.6 percent [15]. In the United States, symptomatic KOA occurs in 6 percent of adults aged 30 years and older and in 10 percent of those aged over 65 years [16, 17]. By comparison, the overall prevalence of KOA in suburban villages of Beijing, China, is approximately 9.6% [18], and a study in Taiyuan showed a prevalence of KOA of 10.9% [19]. The prevalence of KOA was 13.8% in the rural areas of Shanxi Province [20], and a study of urban communities in Heilongjiang showed a prevalence of KOA of 16.1% [10]; our study of rural areas in Tianjin showed a prevalence of KOA of 10.3%. As indicated by the data, there are differences in the prevalence of KOA between these regions; these differences may be related to differences in ethnicity, environmental factors, geography, or lifestyle. Potential biases caused by differences in research methods and demographics may also have some impact on the findings.

The current findings suggest that age is a risk factor for KOA. The prevalence of KOA gradually increased with age and was higher in women than in men. US studies similarly showed that the prevalence of KOA increases with age [16] and that female sex is an independent risk factor for KOA [21]. Furthermore, studies from different regions of China have shown that the prevalence of KOA increases with age, with a higher prevalence among women [10, 19, 20]. Abnormal gait mechanics are implicated in the pathogenesis of KOA. A US study showed that knee kinematics change with age, which may be one of the reasons for the increased prevalence of KOA with age.

Studies have also suggested that kinematic measures have the potential to provide early markers for identifying the risk of developing KOA with age [21]. Additionally, Garcia et al. observed sex differences in gait mechanics and reported that women may have greater joint loads in the medial compartment of the knee [22]. Moreover, evidence suggests that women have altered knee loads compared with men [23] and that sex differences in gait mechanics are one reason for the higher prevalence of KOA in women. In addition, sex differences seem to be one of the main reasons for the risk of KOA. In the present study, after adjusting for other factors, the risk of KOA in postmenopausal women was still 4.12 times that of non-menopausal women. A national study from South Korea showed that the prevalence of OA was higher in women than in men and that the incidence of OA increased significantly during menopause [24]. Another Korean study showed that the increased incidence of postmenopausal OA may be related to female hormonal changes, and the pathogenesis may be that estrogen deficiency affects OA development [25].

The current results also show that frequent walking behavior is an independent risk factor for KOA. With an increase in walking behavior, the prevalence of KOA increases, and frequent walking is an independent risk factor for KOA in men but not in women in our study population. Frequent walkers may have poor walking habits that can lead to long-term damage to the knee joint. A US study has shown that varus thrust during walking is common in patients with KOA and increases the risk of disease progression [26]. However, appropriate physical exercise and correct exercise posture can reduce the prevalence of KOA. Findings from a US study suggest that maintaining physical activity in midlife may have a small but manageable impact on knee function and biomechanics, which translates into a more stable loading environment for the knee in midlife and thus reduces the risk of KOA in future years [27].

Furthermore, a prospective study showed that obesity was an independent influencing factor for KOA [28]. Similarly, research from the US showed that obesity may lead to KOA [29]. Consistent with these findings, the current results show that overweight and obesity are independent risk factors for KOA. However, in the present study population, overweight and obesity were independent influencing factors for KOA in women but not in men. Combined with previous reports, it is speculated that obesity may exert a heavier pressure load on the knee joint compared to normal weight, resulting in a higher prevalence of KOA; however, prospective studies are still needed to determine the impact of a higher load on KOA.

After adjusting for other influencing factors, education level was an independent influencing factor for KOA, and participants with elementary level had the lowest prevalence of KOA. The results of the Korean study showed that lower educational attainment was associated with KOA prevalence and symptoms, indicating that improving socio-educational levels may reduce the socioeconomic burden of KOA [30]. In addition, the results of a meta-analysis showed that educational level can improve the quality-of-life of KOA patients [31]. The reasons for the strong relationship between educational attainment and prevalence of KOA and knee pain are currently unclear, and some unmeasured confounders may have influenced the results.

The prevalence of nocturnal knee pain and sleep problems increases with OA severity, affecting the patients' quality of life [32]. US studies have shown that better sleep quality is associated with reduced knee pain exacerbation [33]. Another US study showed an association between poor sleep and KOA symptoms [34]. Our study found that sleep quality was associated with the prevalence of KOA in both men and women. Although the current analysis of cross-sectional data supports an association between sleep quality and KOA prevalence, this association has not been demonstrated in longitudinal studies. Further research is required to examine the relationship between sleep quality and KOA prevalence.

This study had several limitations. First, this was a cross-sectional study, and we could not clarify any causal relationship between influencing factors in the occurrence and development of KOA. Second, sleep quality was self-reported by the participants through questionnaire and this instrument does not have psychometric properties. Smoking was initially classified into three categories: ever smoker, former smoker, and never smoker. No exposure quantification method was used to adequately summarize a particular individual's smoking history. This may have an impact on the analysis of the factors influencing KOA. Alcohol consumption is similar to that of smoking. In addition, there were no imaging diagnoses in our study, which may have contributed to the decreased prevalence of KOA reported in the text. Finally, some KOA-relative factors, which included sleep conditions, labor intensity, preached cognition, dietary habits, knee related exercise conditions, sun exposure, blood pressure, blood lipid, blood glucose situation and other influence circulating metabolic factors, were lacking in this study. We will continue to monitor the prevalence of KOA among participants in the region in the future. Future studies should include X-ray or MRI examinations to further clarify the causal relationship between KOA influencing factors and KOA prevalence.

## Conclusion

Our study showed that factors such as sex, age, educational level, BMI, sleep quality, and walking frequency were associated with the prevalence of KOA in rural Tianjin. Moreover, there were differences in KOA-related risk factors between sexes. Our study found that risk factors, such as BMI, education level, and menopause, were unique to women. These results suggest that in the future, to prevent the occurrence and development of KOA, sex-specific differences should be considered, especially those for women, who manifest a higher prevalance of KOA and have more risk factors than men. Identifying as many risk factors associated with KOA as possible will help reduce the prevalence of and the socioeconomic burden caused by KOA.

## Data Availability

The datasets generated and/or analyzed during the current study are available from the corresponding author upon reasonable request.

## Abbreviations

OA: Osteoarthritis
BMI: Body mass index
KOA: Knee osteoarthritis
